# HepPar1-Positive Circulating Microparticles Are Increased in Subjects with Hepatocellular Carcinoma and Predict Early Recurrence after Liver Resection

**DOI:** 10.3390/ijms18051043

**Published:** 2017-05-12

**Authors:** Valeria Abbate, Margherita Marcantoni, Felice Giuliante, Fabio M. Vecchio, Ilaria Gatto, Caterina Mele, Antonio Saviano, Damiano Arciuolo, Eleonora Gaetani, Maria C. Ferrari, Igor Giarretta, Francesco Ardito, Laura Riccardi, Alberto Nicoletti, Francesca R. Ponziani, Antonio Gasbarrini, Maurizio Pompili, Roberto Pola

**Affiliations:** 1 Division of Internal Medicine and Gastroenterology, Catholic University School of Medicine, A. Gemelli University Hospital, Rome 00168, Italy; valeriaabbate@libero.it (V.A.); saviano.ant@gmail.com (A.S.); eleonora.gaetani@unicatt.it (E.G.); laura.riccardi71@gmail.com (L.R.); nicstone@hotmail.it (A.N.); francesca.ponziani@yahoo.it (F.R.P.); antonio.gasbarrini@unicatt.it (A.G.); maurizio.pompili@unicatt.it (M.P.); 2 Division of Vascular Medicine, Catholic University School of Medicine, A. Gemelli University Hospital, Rome 00168, Italy; margheritamarcantoni@yahoo.it (M.M.); ilariagatto@yahoo.it (I.G.); ferrari.chiara83@gmail.com (M.C.F.); igorgiarretta@gmail.com (I.G.); 3 Hepatobiliary Surgery Unit, and Catholic University School of Medicine, A. Gemelli University Hospital, Rome 00168, Italy; felice.giuliante@unicatt.it (F.G.); caterina.mele2@gmail.com (C.M.); arditofra@gmail.com (F.A.); 4 Department of Pathology, Catholic University School of Medicine, A. Gemelli University Hospital, Rome 00168, Italy; fabiomaria.vecchio@unicatt.it (F.M.V.); damianoarciuolo@yahoo.it (D.A.)

**Keywords:** microparticles, biomarkers, hepatocellular carcinoma

## Abstract

Circulating microparticles (MPs) are novel potential biomarkers in cancer patients. Their role in hepatocellular carcinoma (HCC) is under intensive investigation. In this study, we tested the hypothesis that MPs expressing the antigen HepPar1 are increased in the blood of subjects with HCC and may serve as markers of early recurrence after liver resection (LR). We studied 15 patients affected by HCC undergoing LR, and used flow cytometry to assess the number of circulating HepPar1+ MPs. Ten subjects without HCC (five with liver cirrhosis and five with healthy livers) were used as controls. After LR, HCC patients underwent a follow-up to check for early recurrence, which occurred in seven cases. The number of circulating HepPar1+ MPs was significantly higher in subjects affected by HCC, compared to individuals without cancer (*p* < 0.01). We also found that, among HCC patients, the number of circulating HepPar1+ MPs, measured before LR, was significantly higher in those who displayed early recurrence compared to those without recurrence (*p* = 0.02). Of note, other types of circulating MPs, such as those derived from endothelial cells (CD144+) or those produced by the activated endothelium (CD144+/CD62+), were not associated with HCC, nor could they predict HCC recurrence. HepPar1+ MPs deserve further investigation as novel biomarkers of disease and prognosis in HCC patients.

## 1. Introduction

Hepatocellular carcinoma (HCC) accounts for more than 90% of primary liver cancers and is the fifth most common type of cancer and the second leading cause of cancer-related deaths worldwide [1]. When possible, liver resection (LR) is the first choice of treatment [2,3,4]. However, long-term results demonstrate that HCC recurrence after LR may be as high as 70–85% in five years. For this reason, the survival rate is only 30–50% over the same period of time [5,6]. When HCC recurs more than two years after LR, this is mainly due to the emergence of new neoplastic lesions, as a consequence of the carcinogenic environment of the remnant liver [7,8,9]. However, recurrence after LR may occur earlier (within two years). It is commonly accepted that this phenomenon depends on the intrinsic aggressive biology of the tumor and/or the presence of microscopic intrahepatic metastases that were undetectable at the time of LR. Several studies have tried to identify predictors of early recurrence [10,11,12,13,14,15,16,17]. Nonetheless, a reliable predictor of HCC recurrence is still lacking.

Microparticles (MPs) are a type of extracellular vesicles produced by cells upon activation, stimulation, and death. They are between 100 and 1000 nm in size and bear on their surface the antigenic markers of the parent cell, since they are produced through budding of the plasma membrane, the formation of membrane “blebs”, and eventual release into the circulation. While once considered just inert remnants of cellular processes, MPs are now recognized as important players in many physiological and pathological conditions, due to the fact that they act as cargos for nucleic acids and proteins and have the ability to translate functionally important biological information between cells. In the last few years, increasing attention has been paid the possible use of circulating MPs as novel biomarkers of disease, including in the field of hepatology [18,19,20,21,22,23].

In this study, we have evaluated whether MPs expressing the antigen HepPar1 may serve as biological markers of HCC and early HCC recurrence after LR.

## 2. Results

Fifteen patients with HCC were enrolled in the study. For a control, we also studied 10 patients without HCC. Of these, five had liver cirrhosis and five had healthy livers. The main demographical, clinical, laboratory, and histological features of the studied population are reported in Table 1.

Among the HCC patients, twelve were male and three were female. The median age was 70 years (range 35–83 years). The median HCC size was 4.5 cm (range 1–16 cm). Eight patients had liver cirrhosis, due to either hepatitis C virus (four cases), alcohol abuse (two cases), hepatitis B virus (one case), or non-alcoholic steatohepatitis (one case). Five patients had no cirrhosis, but were affected by either non-alcoholic steatohepatitis (four cases) or hepatitis B infection (one case). Two patients had HCC on a healthy liver. Among the subjects without HCC, five had liver cirrhosis and five had a healthy liver. The subjects with cirrhosis had a median age of 67 years (range 60–72 years), and consisted of three males and two females. Cirrhosis was due to either hepatitis C virus (two cases), hepatitis B virus (two cases), or due to cryptogenic cirrhosis (one case). The subjects with healthy livers had a median age of 73 years (range 69–78 years), and consisted of three males and two females.

Table 2 presents the number of circulating HepPar1+ MPs measured in HCC patients and subjects without HCC. In the HCC population, the median number of HepPar1+ MPs was 283/µL of blood (10–3865 MPs/µL). This was significantly higher than the number of HepPar1+ MPs circulating in the blood of subjects with only liver cirrhosis or healthy livers (*p* < 0.01). Notably, such a difference between the groups did not exist when other types of circulating MPs (CD144+ and CD144+/CD62E+) were analyzed.

Next, we focused on subjects with HCC undergoing LR. Among them, four were treated with major resection (≥3 liver segments) and 11 were treated with minor resection (anatomical in seven cases and non-anatomical in four cases), as described [24]. In all cases, R0 resection was performed, with the margin of resection ranging from 1 to 25 mm. No deaths occurred during surgery. Postoperative morbidity was defined according to the Dindo Clavien classification [25], and involved 5/15 patients (33%, grade ≥3 in three cases).

Postoperative pathology showed the following. In 11 cases, resected tumors were highly or moderately differentiated. Two patients had mixed hepatocellular-cholangiocarcinoma with moderate differentiation of the HCC component (grading G2). Eight patients had microvascular invasion. Seven patients had capsular invasion. Two patients had tumor satellitosis. The rate of Ki67 positivity results were low in two patients, medium in seven patients, and high in three patients. It was not tested in three cases due to technical reasons.

Postoperative follow-up started the day after LR. No patients died or were lost during follow-up. HCC recurrence was observed in seven patients (46.7%). In all of these cases, recurrence was intrahepatic and was detected within 12 months after LR. It was multifocal in one case, occurred in a liver segment next to the resected one in three cases, and occurred in the controlateral liver lobe in the remaining three cases. Among the patients who presented recurrence, four had liver cirrhosis (Hepatitis C virus-related in two cases, steatohepatitic in one case and alcohol-related in the remaining case), two were affected by non-alcoholic steatohepatitis, and one had a healthy liver. In the eight patients who did not display recurrence, the median follow-up was 21 months (range 16–26 months).

Table 3 presents the demographical, clinical, laboratory, and histological features of the subjects with and without early HCC recurrence.

The only significant difference found between subjects with and without recurrence was age (*p* = 0.04), while gender, presence of cirrhosis, levels of α-feto protein (AFP), Aspartate aminotransferase (AST), and Alanine transaminase (ALT), tumor size, margin-free width, tumor grading, satellitosis, capsular invasion, microvascular invasion, and percentage of Ki67-positive cells did not show statistically significant association with tumor recurrence.

**Figure 1:**
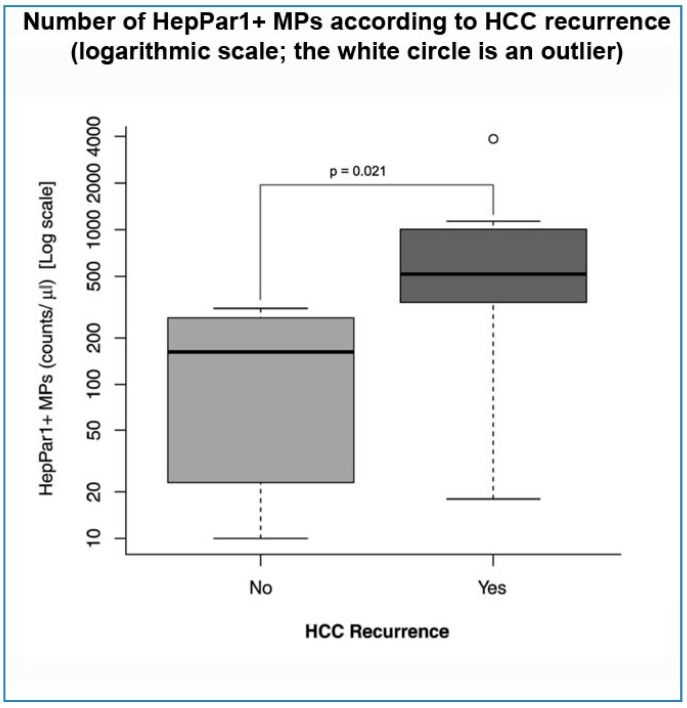
Number of HepPar1+ microparticles (MPs) according to hepatocellular carcinoma (HCC) recurrence.

This was not the case when the number of circulating HepPar1+ MPs, measured before LR, was analyzed. Indeed, these MPs were significantly more numerous in the blood of subjects who displayed recurrence, compared to those who remained cancer-free during follow-up (*p* = 0.02) (Table 4 and Figure 1). Of note, such a difference was not found when other circulating MPs (CD144+ and CD144+/CD62E+) were analyzed (Table 4).

**Figure 2:**
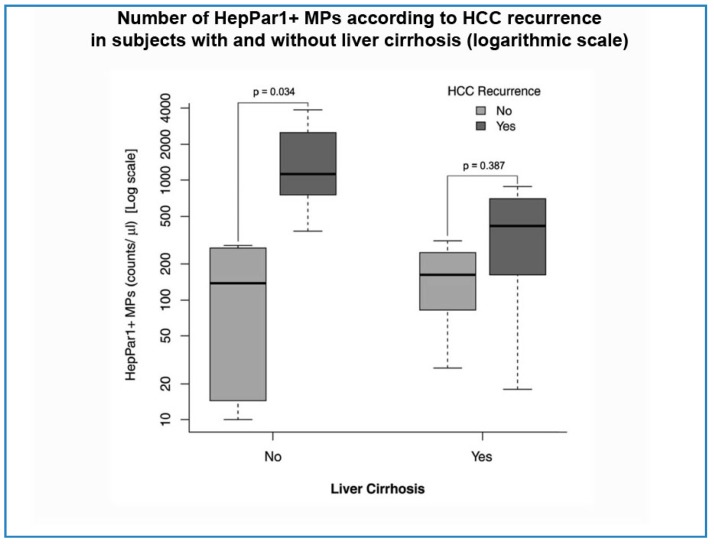
Number of HepPar1+ MPs according to HCC recurrence in subject with and without liver cirrhosis.

Based on the fact that HepPar1+ MPs do not seem to increase in subjects with cirrhosis but without HCC (as shown in Table 2), we decided to separately analyze HCC subjects with and without liver cirrhosis. In particular, we looked at the number of HepPar+ MPs in HCC subjects with and without cirrhosis, who presented or did not present HCC recurrence after LR. Both in the absence and the presence of cirrhosis, the number of these MPs was higher in patients with recurrence (1133 vs. 137.5/µL and 441.5 vs. 162/µL, respectively). However, this difference was statistically significant only in the subgroup of patients who had no cirrhosis (*p* = 0.03) (Figure 2).

**Figure 3:**
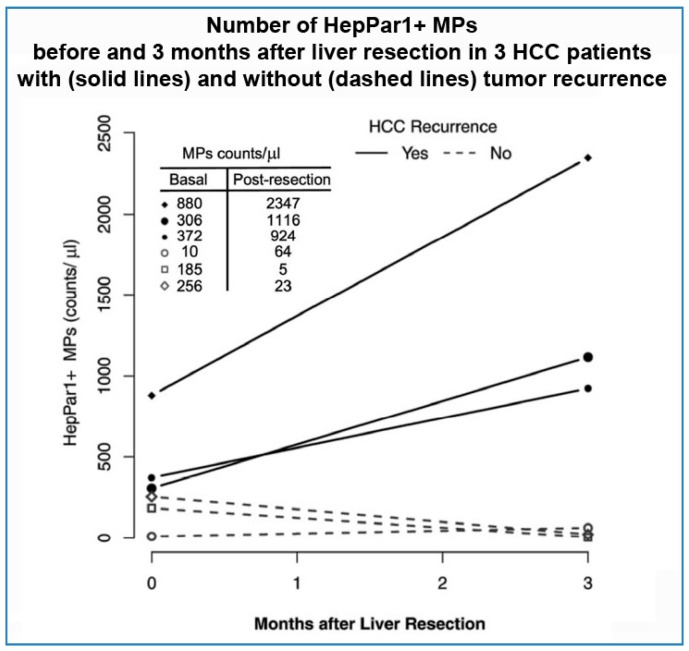
Number of HepPar1+ MPs before and 3 months after liver resection on 3 HCC patients with (solid lines) and without (dashes lines) tumor recurrence.

We did not carry out a formal time-course assay of circulating HepPar1+ MPs after LR. However, in six patients, a second assessment of the number of circulating HepPar1+ MPs was conducted three months after LR. Of these six patients, three presented HCC recurrence at later time-points of the follow-up, while the other three remained free from recurrence for the entire follow-up period. Interestingly, in the three cases that later presented recurrence, the number of HepPar1+ MPs was sharply higher at three months after LR than before surgery. In contrast, in the three patients who did not present recurrence, the number of HepPar1+ MPs assessed three months after LR was either lower than that measured before surgery (in two cases) or only slightly increased (in one case) (Figure 3).

## 3. Discussion

This is a pilot study showing that assessing the number of HepPar1+ MPs in the blood of subjects with unifocal surgically resectable HCC may help to identify those patients who will have early tumor recurrence after surgery. Another novel finding of this study is that HepPar1+ MPs are virtually absent in the circulation of subjects without HCC, even if affected by liver cirrhosis. Likewise, they are barely detectable in the blood of subjects with healthy livers. These data are important, since they indicate that circulating HepPar1+ MPs are tumor-specific and may have the potential to serve as biomarkers for the diagnosis of HCC. This is consistent with the fact that HepPar is an antigen used for the staining of HCC [19], that HepPar1+ MPs have already been associated with tumor size in patients affected by HCC [26], and that HepPar1+ MPs decrease after HCC removal by means of liver transplantation [26].

Regarding the association of HepPar1+ MPs with early HCC recurrence, this might be explained by hypothesizing that HepPar1+ MPs are abundant in blood circulation when microscopic undetected tumor foci exist in the liver, in addition to the main tumor that will be removed at surgery. In this model, early recurrence would be driven by the activation of such unremoved additional tumor foci. Consistent with this hypothesis is the fact that, in our study group, recurrence was either multifocal, or occurred in liver segments different from those in which the primary tumor had originated. This hypothesis is also reinforced by the finding that patients with early recurrence displayed an increased number of circulating HepPar1+ MPs three months after LR. In contrast, no early recurrence occurred among subjects who displayed decreased or substantially unchanged number of HepPar1+ MPs three months after LR. Although the numbers of subjects included in this analysis is low, this is an interesting observation which deserves further investigation in future studies.

An additional interesting finding of this study is that the association between HepPar1+ MPs and early HCC recurrence is particularly strong in non-cirrhotic patients. The reasons underlying this phenomenon remain to be elucidated. However, this might be important for specific categories of patients, such as those with non-alcoholic fatty liver disease, in whom up to 50% of HCC occurs in the absence of cirrhosis [27].

In this study, in addition to HepPar1+ MPs, we also assessed the number of other types of circulating MPs, i.e., those produced by endothelial cells (CD144+) and activated endothelial cells (CD144+/CD62E+). We decided to look at these MPs because vascular invasion, angiogenesis, and endothelial activation have been previously proposed as potentially important modulators of HCC pathogenesis, invasiveness, and prognosis. Nonetheless, these MPs did not shown any significant association with HCC presence or early recurrence after LR. This is important, since it strengthens the concept that organ- and disease-specific MPs should be studied when looking for biomarkers with diagnostic and prognostic value. A recent example has been provided by Julich-Haertel and colleagues, who have found that MPs positive for AnnexinV, EpCAM, and ASGPR1 may help distinguish between subjects with liver cancer and subjects with cirrhosis but no detectable liver malignancy [20].

Apart from HepPar1+ MPs, among all the demographical, clinical, surgical, and histological parameters that we took into account in this study, only younger age showed a mildly significant association with HCC recurrence. This is consistent with previous studies that have associated young age with poorer short- and long-term prognosis in HCC patients [28,29]. However, these findings must be considered with caution, since other studies have found an association between early HCC recurrence and parameters such as tumor size, microvascular invasion, tumor grading and width of the resection margins [7,17]. Our small sample size might have had an effect on these findings.

## 4. Materials and Methods

### 4.1. Patients

We enrolled patients with a novel diagnosis of unifocal surgically resectable HCC admitted to the Hepato-Biliary Surgery Unit of the A. Gemelli University Hospital of Rome, Rome, Italy, from January 2013 to December 2015. In some cases, patients were addressed to the Hepato-Biliary Surgery Unit upon identification of HCC during a regular surveillance program for liver cirrhosis at the Outpatient Liver Unit of the Agostino Gemelli University Hospital. In other cases, patients were diagnosed with HCC occasionally, or because of the presence of overt tumor-related clinical signs and symptoms. This second type of subject was not necessarily affected by cirrhosis or other known chronic liver diseases. In all cases, the diagnosis of HCC was made by ultrasonography (US), computed tomography (CT), and/or magnetic resonance imaging (MRI), according to current international guidelines [2,3,4]. US-guided liver biopsy was reserved to tumors complicating cirrhosis with atypical dynamic behavior after injection of contrast enhancement or to tumors emerging in non-cirrhotic livers. All patients underwent bone scintigraphy and body CT scan before LR in order to exclude the presence of extrahepatic disease. Subjects with recurrent HCC after a previous curative treatment or with clinical history of other neoplasms were excluded. Cirrhotic patients without HCC were enrolled among those admitted to the Liver Unit of the Agostino Gemelli University Hospital during March 2017. All patients with cirrhosis (with or without HCC) enrolled in the study had preserved liver function (Child-Pugh Class A). Subjects with healthy livers were healthy volunteers enrolled during March 2017 at the Agostino Gemelli University Hospital. All subjects gave informed consent to participate in the study, which was approved by the Review Board of the Institute of Special Surgical Pathology, (Istituto di Patologia Speciale Chirurgica) of the Catholic University School of Medicine on 18 July 2012 (document no. 22) and was conducted in conformity to the 1990 Declaration of Helsinki and successive amendments.

### 4.2. Liver Resection

All patients fulfilled the following criteria: they exhibited (1) bilirubin levels less than 2.0 mg/dL; (2) an absence of ascites and macrovascular intrahepatic invasion on imaging methods; and (3) their remnant liver volume was more than 50% of their total liver volume. Extension of liver resection was decided according to the result of Indocyanine Green Clearance test [30], which was performed in all patients; patients with 15-min retention rates ≥10% were excluded from major resection. In all patients the absence of other focal liver lesions, apart from the resected tumor and macroscopic vascular invasion, was confirmed by intraoperative US with contrast medium (SonoVue^®^, Bracco Imaging Spa, Milano, Italy). In all patients, control of hepatic pedicle was obtained beforehand and intermittent pedicle clamping was used in 80% of the patients. Parenchymal transection was performed using the wet bipolar forceps and CUSA (Cavitron Ultrasonic Surgical Aspirator System 200; Valleylab, Boulder, CO, USA). Hemostasis and biliostasis were obtained with absorbable clips (Absolok Extra AP200 and AP300, Ethicon Endo-Surgery Inc., Somerville, NJ, USA) and absorbable sutures [31]. After LR, patients entered a surveillance program for the detection of early HCC recurrence. Medical evaluations were carried out every three months, along with laboratory tests, liver US and AFP determination. Independently from the results of the liver US, all patients underwent contrast enhanced abdomen CT scans within six months of LR, and then at six-month intervals. Every new focal lesion detected on US was characterized using CT or MRI and treated according to the current management guidelines of HCC [2,3,4].

### 4.3. Histological Data

Resected specimens were analyzed with histological and immunohistochemical techniques in order to determine: (1) tumor grading according to Edmondson and Steiner [32]; (2) Ki67 Index [33]; (3) microvascular invasion, defined as the invasion of portal vein branches in portal tracts, central veins in noncancerous liver tissue and venous vessels in the tumor capsule or non-capsular fibrous septa [34]; (4) capsular infiltration, defined as the presence of tumor cells within the capsule; and (5) peritumoral satellitosis, that is, the presence of satellite peritumoral nodules.

### 4.4. Analysis of microparticles (MPs)

**Figure 4:**
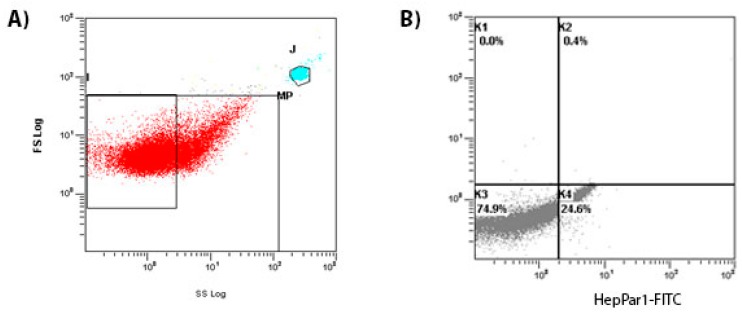
Circulating MPs are represented on a forward scatter/side scatter dot plot. (**A**) Total MPs are defined as events with a size of 0.1 to 1.0 µm gated in the window using 1-µm diameter calibrant beads as the interior criterion prior to the sample testing; (**B**) Size-selected events are plotted according to their fluorescence for specific HepPar1 binding on a fluorescence plot. Events included in the K4 section were considered HepPar1+ MPs. J is the gate of definition of fluorescent megamix beads.

Blood was collected into citrate vacutainer tubes from a peripheral vein using a 21-gauge needle and processed immediately. Samples were centrifuged at 450 g for 20 s at room temperature to collect platelet-rich plasma (PRP) and then at 1500 g for 20 s to generate platelet-free plasma (PFP). MPs of hepatic and endothelial origins were characterized according to the expression of membrane-specific antigens. To identify hepatic MPs, 150 µL of PFP were incubated with unconjugated mouse anti-human HepPar1 antibody (DAKO) for 1 h in the dark at room temperature at 1:50 dilution, as recommended by the manufacturer. Secondary goat anti-mouse IgG fluorescein isothiocyanate (FITC)-conjugated antibody (Thermo Fisher Scientific, Waltham, MA USA) was added to the samples at 1:100 dilution and incubated for additional 30 min. In order to determine endothelial and activated endothelial MPs, 150 µL of PFP were incubated for 30’ in the dark at room temperature with 3 µL of FITC-labeled mouse anti-human CD144 (BD Pharmingen, Milan, Italy) and 1.5 µL of phycoerythrin (PE)-labeled mouse anti-human CD62E (BD Pharmingen). An equal volume of Flow Count fluorospheres (Beckman Coulter, Milan, Italy) was added to the samples in order to determine MPs concentration, and all of the samples were analyzed by a FC500 Flow Cytometer (Beckman Coulter). The fluorescent Megamix beads (Biocytex, Marseille, France) covering the MPs (0.5 and 0.9 µm) and platelet size ranges (0.9 and 3 µm) were used for size calibration. A total of 150,000 events were acquired for each sample; values are reported as number of MPs in 1 µL of PFP (number/µL). Representative plots of our cytofluorimetric analyses are presented in Figure 4.

### 4.5. Statistical Analysis

Continuous variables were expressed as median, range and quartiles (Q1 = 25th percentile, Q3 = 75th percentile); quantitative variables were expressed as absolute numbers and percentages. Correlation and association between HepPar1+ MPs and HCC were evaluated with the Spearman coefficient, the Mann-Whitney *U* test and the Kruskal Wallis test. Correlation and association between pre-treatment variables and HCC recurrence were tested with the Mann-Whitney *U* test, Kruskal Wallis test and the Fisher exact test. We considered as statistically significant values of *p* < 0.05. The statistical analysis was performed by software. IBM SPSS Statistics for Windows, Version 20.0, released 2011 (IBM Corp., Armonk, NY, USA) and R Statistics version 3.2.3 (R Foundation for Statistical Computing, Wine, Austria. ISBN 3-900051-07-0, URL http://www.R-project.org).

## 5. Conclusions

There is an urgent need for the identification of non-invasive diagnostic markers for HCC and biological predictors of early HCC recurrence after surgery. Our study shows that MPs carrying the antigen HepPar1 are novel candidates to serve these purposes and merit further investigation for their potential role in the diagnosis, prognosis, and follow-up of subjects with liver cancer.

## Figures and Tables

**Table 1:** Demographical, clinical, laboratory, and histological characteristics of the studied population.

Variables		HCC (*n* = 15)	No HCC (*n* = 10)	
			Liver Cirrhosis (*n* = 5)	Healthy Liver (*n* = 5)
Male (%)		12 (80.0)	3 (60.0)	3 (60.0)
Age (years)		70 (35–83)	67 (60–72)	73 (69–78)
Cirrhosis (%)		8 (53.3)	5 (100.0)	0 (0.0)
AFP (ng/mL)		4 (1–4449)	3.5 (2.4–15)	N/A
AST (UI/l)		33 (19–87)	24 (24–46)	11 (9–15)
ALT (UI/l)		28 (11–86)	24 (15–34)	10 (8–15)
HCC Size (cm)		4.5 (1–16)	-	-
Grading	G1 (%)	2 (13.3)		
	G2 (%)	9 (60.0)	-	-
	G3 (%)	4 (26.7)		
Satellitosis (%)		2 (13.3)	-	-
Capsular invasion (%)		7 (46.7)	-	-
Microvascular invasion (%)		8 (53.3)	-	-
Ki67	Low (%)	2/12 (16.7)		
	Medium (%)	7/12 (58.3)	-	-
	High (%)	3/12 (25.0)		
Margin-free width (mm)		5 (1–25)	-	-

HCC: Hepatocellular carcinoma; AFP: α-feto protein; AST: Aspartate aminotransferase; ALT: Alanine transaminase.

**Table 2:** Number of circulating MPs in HCC patients and subjects without HCC.

Circulating MPs	HCC (*n* = 15)	No HCC (*n* = 10)		*p*-Value
		Liver Cirrhosis (*n* = 5)	Healthy Liver (*n* = 5)	
HepPar1+ MPs/µL	283 (10–3865)	7 (3–10)	6 (3–7)	<0.01
CD144+ MPs/µL	25 (18–48)	22 (14–39)	20 (14–41)	n.s.
CD144+/CD62E+ MPs/µL	8 (3–17)	8 (4–15)	6 (3–18)	n.s.

n.s.: Not significant.

**Table 3:** Demographical, clinical, laboratory, and histological characteristics of subjects with and without early HCC recurrence.

Variables		HCC Recurrence (*n* = 7)	No Recurrence (*n* = 8)	*p*-Value
Male (%)		5 (71.4)	7 (87.5)	n.s.
Age (years)		56 (35–78)	73 (62–83)	0.04
Cirrhosis (%)		4 (57.1)	4 (50.0)	n.s.
AFP (ng/mL)		9.5 (1–4449)	3.9 (2–4)	n.s.
AST (UI/L)		36 (19–87)	32.5 (19–48)	n.s.
ALT (UI/L)		28 (11–86)	33.5 (19–55)	n.s.
HCC Size (cm)		4.0 (1–16)	4.8 (2.4–10)	n.s.
Grading	G1 (%)	1 (14.3)	1 (12.5)	
	G2 (%)	6 (85.7)	3 (37.5)	n.s.
	G3 (%)	0 (0.0)	4 (50.0)	
Satellitosis (%)		1 (14.3)	1 (12.5)	n.s.
Capsular invasion (%)		4 (57.1)	3 (37.5)	n.s.
Microvascular invasion (%)		4 (57.1)	4 (50.0)	n.s.
Ki67	Low (%)	1 (16.7)	1 (16.7)	
	Medium (%)	4 (66.7)	3 (50.0)	n.s.
	High (%)	1 (16.7)	2 (33.3)	
Margin-free width (mm)		2 (1–25)	6 (1–12)	n.s.

**Table 4:** Circulating MPs in HCC patients with and without recurrence.

Circulating MPs	HCC Recurrence (*n* = 7)	No Recurrence (*n* = 8)	*p*-Value
HepPar1+ MPs/µL	517 (18–3865)	162 (10–310)	0.02
CD144+ MPs/µL	23 (19–34)	26 (18–48)	n.s.
CD144+/CD62E+ MPs/µL	7 (3–17)	9 (4–16)	n.s.
